# Endoscopic posterior vault distraction osteogenesis (PVDO): advantages of eliminating the bicoronal incision

**DOI:** 10.1007/s00381-026-07391-w

**Published:** 2026-07-23

**Authors:** Philip D. Tolley, Maura R. Guyler, Isabel A. Ryan, Elizabeth B. Card, Adam J. Kundishora, Jordan W. Swanson

**Affiliations:** 1 Division of Plastic, Reconstructive, and Oral Surgery, Children’s Hospital of Philadelphia, Philadelphia, PA USA; 2 Division of Neurosurgery, Children’s Hospital of Philadelphia, Philadelphia, PA USA; 3 Department of Neurosurgery, University of Pennsylvania Perelman School of Medicine, Philadelphia, PA USA

**Keywords:** Cranial morphology, Craniosynostosis, PVDO

## Abstract

Cranial vault modeling for children with craniosynostosis is traditionally performed utilizing an open bicoronal approach. For patients with multi-suture craniosynostosis, particularly bilateral coronal suture fusion, posterior vault expansion using cranial springs or distractors is increasingly utilized. Posterior vault distraction osteogenesis (PVDO) enables substantial expansion of intracranial volume, minimizes relapse, and corrects cranial dysmorphology. However, the burden of a large bicoronal scar remains challenging for patients and families. Minimally invasive endoscopic approaches used primarily for single-suture craniosynostosis allow for decreased blood loss, shorter hospital stay, and decreased scar burden. However, minimally invasive techniques have not yet been realized for PVDO, for several reasons including the safe navigation of venous anatomy. In this report, the authors describe a novel technique utilizing a minimally invasive endoscopic PVDO technique (PV En-DO) that avoids a bicoronal incision and present an initial case report demonstrating feasibility in patients with bicoronal craniosynostosis. The authors conclude that PV En-DO appears feasible, safe, and effective, offering adequate cranial vault expansion with reduced scar burden and advantageous perioperative characteristics. Further study is needed to elucidate the indications, safety, and long-term outcomes of this technique.

Posterior vault distraction osteogenesis (PVDO) increases intracranial volume and improves cranial morphology in patients with craniosynostosis [1–4]. Since its introduction by White et al. in 2009 [5], evidence supporting its safety, effectiveness, and indications has grown [1, 6, 7]. However, bicoronal scarring after cranial vault surgery remains a concern for patients and families [8–10]. Minimal incision techniques for single-suture craniosynostosis have been shown to decrease blood loss, scarring, and length of stay [11–13]. Endoscopic-assisted fronto-orbital distraction osteogenesis (Endo-FODO) serves as a technique precedent for the anterior vault, with several advantages over open techniques [14–16]. Minimally invasive approaches to PVDO, however, have not been described. In this study, we present a novel endoscopic approach to PVDO (PV En-DO) and a patient report demonstrating its role in treating bicoronal craniosynostosis.

## Technique

**Fig. 1:**
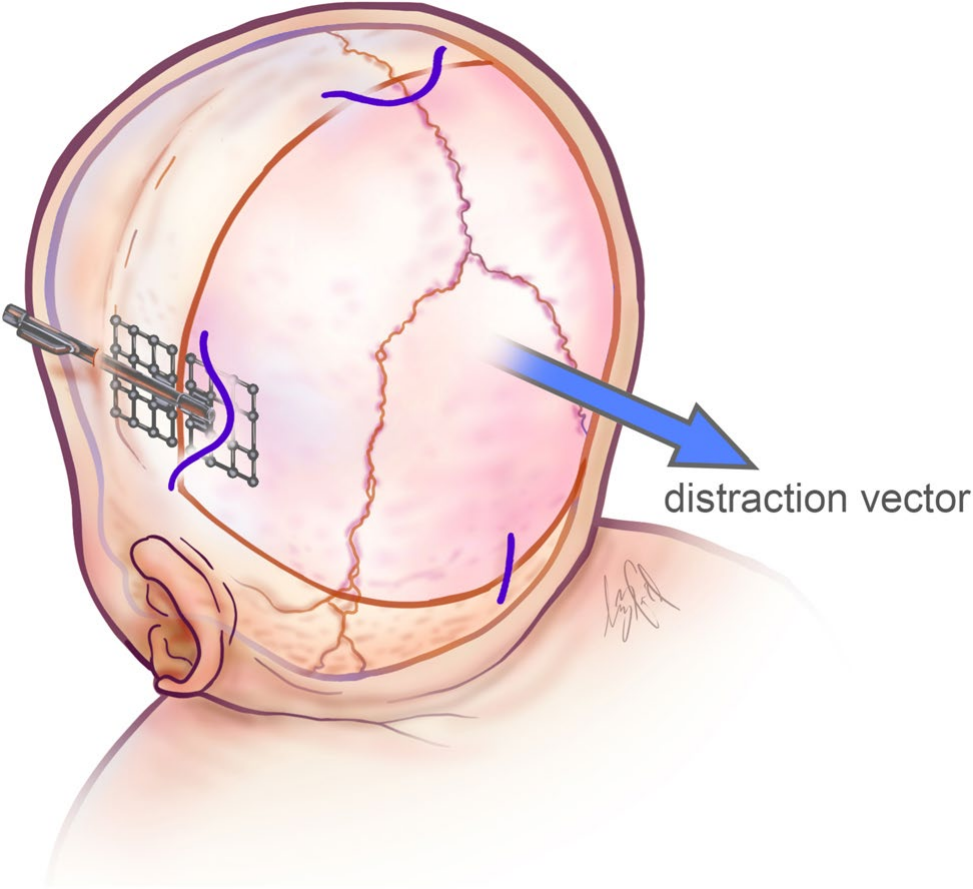
Illustration of minimally invasive incision pattern and osteotomy location

After induction of general anesthesia, the patient is placed prone. Two 3-cm rounded chevron incisions are designed at each end of a bicoronal incision in the parietal scalp, with a 2.5-cm chevron incision at the midline vertex, and a 1-cm vertical incision in the low occipital hairline (Fig. 1).

A narrow hair shave is performed, local anesthesia is infiltrated, distractor positions are planned, and the head is prepped.

A fifteen blade is used to incise all four incisions, and needlepoint electrocautery is used to divide the galea. Using a 30-degree endoscope, extended-length blunt-tipped protected cautery is used to create subgaleal tunnels between the four incisions and over the planned craniotomies. These are in the coronal plane vertically at the region of greatest biparietal width, and in a downward-slanting axial plane in the low occipital region, 2-cm inferior to the inion, meeting at an approximate 100-degree angle. Through the lateral incisions, the posterior margin of the temporalis muscles are elevated. The pericranium is linearly incised and stripped narrowly over the planned craniotomies, such that the distractor footplates are placed beneath periosteal flaps. Four total burr holes are made at the vertex, midline occiput, and bilateral parietal osteotomy intersects. A Hudson Brace is used to initiate the craniotomies; resected bone is saved for particulate graft. Penfield 3 elevators are used for epidural dissection beneath scored osteotomies. Under 0- and 30-degree endoscope guidance, a piezoelectric saw (Mectron, Milan, Italy) is used to make submillimeter craniotomies medially to laterally. A Gigli saw-type malleable retractor is placed in the epidural space to protect the underlying dura and sinuses.

After confirmed segment mobility, bilateral cranial distractors (KLS Martin, Baden-Württemberg, Germany) are placed in the bilateral low parietal regions. Gelfoam (Pfizer Inc., New York, NY) is placed in 2 × 4-cm pads in the epidural space beneath the planned distractor sites for further dural protection. The distractor vector is slightly downward, and parallel to prevent interference. Activating arms exit anteriorly through a small percutaneous incision and are delivered with red rubber catheters. The distractors are fixated with resorbable Injectable Polymer System (IPS) fixators (DePuy Synthes, West Chester, PA) activated to verify mobility, closed completely, and ratchet mechanisms are engaged. The saved particulate bone is placed in the burr holes and stabilized with Surgiflo (Ethicon Inc, Cincinnati, OH). The bilateral temporal muscle flaps are inset over the distractors to the pericranial flaps to restore pericranial continuity and facilitate ossification. The incisions are closed in two layers with absorbable suture and a headwrap is placed. Distraction begins on post-operative day (POD) two at rate of 1 mm/day and rhythm of twice daily, and continues until adequate volume expansion is achieved, as determined clinically and radiographically.

### Case report

**Fig. 2:**
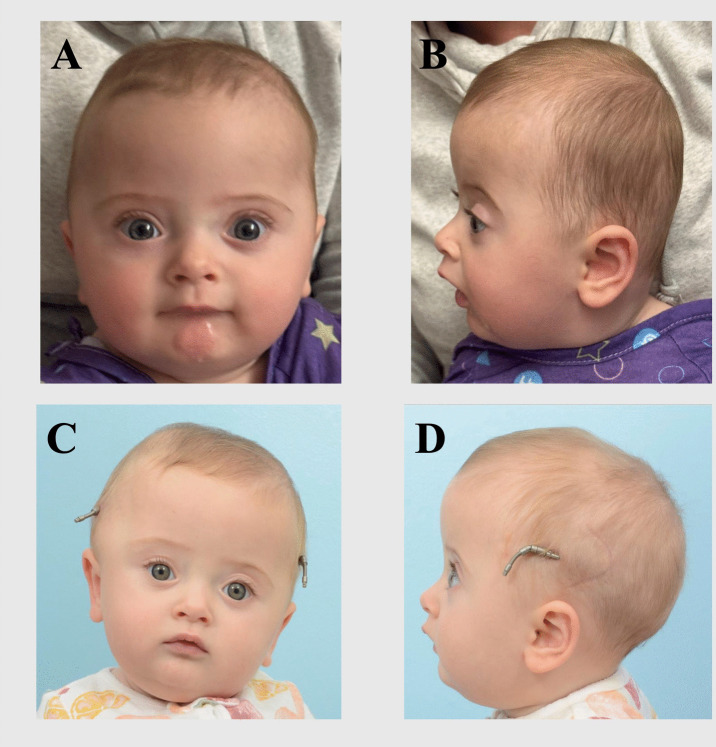
**A**, **B** Mild turribrachycephaly, mild to moderate frontal bossing and supraorbital retrusion, pictured here pre-operatively. (**C**, **D**) one month post-operative from endoscopic-assisted posterior vault distraction osteogenesis, at consolidation

**Fig. 3:**
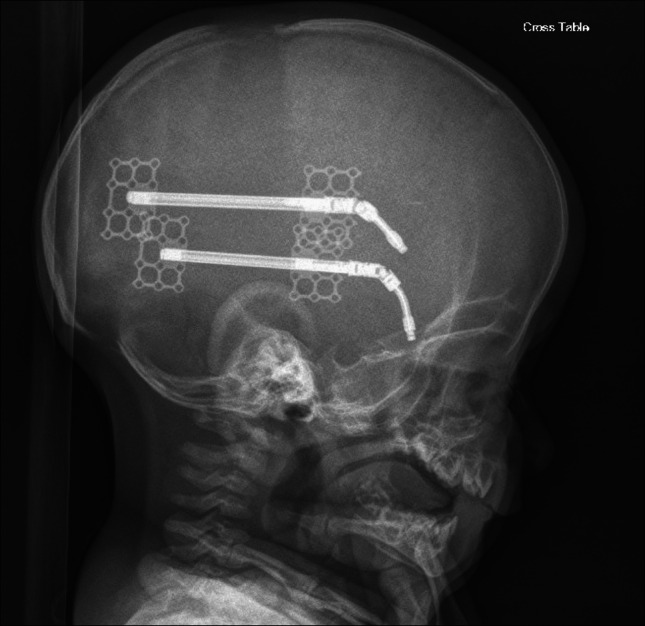
Lateral radiograph taken at consolidation showing robust posterior cranial vault expansion

A 4-month-old female presented with turribrachycephaly, moderate frontal bossing, and supraorbital retrusion. Cephalic index was 0.99, with 3 mm of axial cranial vault asymmetry. CT imaging demonstrated bicoronal and partial left squamosal craniosynostosis, without evidence of elevated ICP. Preoperative ophthalmologic exam was negative for papilledema. Physical exam and rapid craniosynostosis panel were negative for syndromic diagnoses. PV En-DO was performed at 6-months-old. Operative time was 128 min, with 50 mL EBL and no blood transfusion. She was discharged on POD2 after distractor activation began. Activation lasted for 37 days and showed 35 mm radiographic advancement (Figs. 2 and 3).

## Discussion

We describe an endoscopic approach to posterior vault distraction osteogenesis (PV En-DO) to treat bicoronal craniosynostosis. In this report, PV En-DO was feasible, safe, and effective, achieving adequate vault expansion with minimal scarring. EBL and surgical duration were lower than typically reported for PVDO, consistent with perioperative benefits seen in Endo-FODO [2, 15].

Replacing the bicoronal incision with four small incisions reduced scar burden, a significant parental concern, with risks of alopecia and scar asymmetry being of distress [8–10, 17]. While early PVDO is associated with a decreased need for frontofacial advancement among patients with syndromic craniosynostosis, many still require it [7, 15]. In those likely to need future bicoronal access, the benefits of avoiding the incision at the time of PVDO are less evident. Patient selection is therefore critical. This patient had no findings suggestive of a syndromic diagnosis and was considered unlikely to require future anterior vault surgery.

Success hinges on several technical points. First, we employ a low posterior cranial osteotomy below the level of the torcula, which increases intracranial volumetric gains without significant additional complications [4]. Second, incision placement allows direct visualization of the four burr holes, enabling epidural dissection of planned craniotomy vectors. While interdigitating parietal bone flaps are used in PVDO to facilitate stability of the regenerate, a linear coronal craniotomy is performed in the PV En-DO, which may impact stability. However, high quality ossification in this patient population should mitigate this risk. Resorbable IPS fixation of distractor footplates rather than titanium fixation, allows easier removal through smaller incisions [18]. Finally, endoscopy and malleable epidural retractors minimize risk of dural or sinus injury, particularly of the transverse sinus, which is proximal and parallel to the low parietal craniotomy. A low threshold for conversion to open exposure is maintained if dural or vascular injury occurs.

These early results suggest PV En-DO is safe, feasible, and may decrease scar burden and cranial devascularization. We advocate for careful patient selection of those unlikely to require future bicoronal access. Further study is warranted to define the indications and safety profile of PV En-DO.

## Data Availability

No datasets were generated or analysed during the current study.
